# Roles of Electrical Impedance Tomography in Determining a Lung Protective Strategy for Acute Respiratory Distress Syndrome in the Era of Coronavirus Disease 2019

**DOI:** 10.31662/jmaj.2021-0014

**Published:** 2021-04-02

**Authors:** Toru Kotani, Atsuko Shono

**Affiliations:** 1 Department of Intensive Care Medicine, Showa University School of Medicine, Tokyo, Japan

**Keywords:** electrical impedance tomography, coronavirus disease 2019, acute respiratory distress syndrome, lung protective strategy

## Abstract

Electrical impedance tomography (EIT) is noninvasive and can be used at the bedside for real-time evaluation to identify ventilation distribution of infected lungs. This review briefly describes the basic principle of EIT and summarizes the latest findings on its potential contribution to lung protective strategies in coronavirus disease 2019 patients. Additionally, experimental approaches for detecting the distribution of pulmonary blood flow in coronavirus disease 2019 patients are presented. The findings underscore the role of EIT in determining lung protective strategies for coronavirus disease 2019-associated acute respiratory distress syndrome.

## Introduction

Acute respiratory distress syndrome (ARDS) is characterized by life-threatening hypoxemia caused by hyperpermeability of pulmonary capillaries resulting from various background diseases or conditions. Although mechanical ventilation is required for ARDS diagnosis and treatment, it has become obvious that ventilator-associated lung injury plays an important role in the development of multiple organ failure, which is the cause of death for ARDS. Large-scale clinical trials have demonstrated that low tidal volume ventilation (LTV), which limits both tidal volume and plateau pressure, improves ARDS prognosis. Currently, LTV is one of lung protection strategies and is essential for treating ARDS.

When gas exchange cannot be maintained by LTV, early implementation of prone positioning acts as rescue therapy and improves the prognosis. It is well known that prone positioning recruits collapsed regions and improves oxygenation. Recent studies have suggested that prone positioning promotes homogeneous ventilation distribution and might have a lung protective effect. If the lungs are evenly ventilated by reducing regional overdistention and recruiting the collapsed region, local stress and strain can be ameliorated, resulting in the prevention of ventilator-induced lung injury. The importance of homogeneity of regional ventilation has been increasingly recognized. It has been reported that lung inhomogeneity during tidal ventilation increases dynamic lung strain ^(1)^. In an animal model, increased dynamic strain was more associated with the development of pulmonary edema, derangement of lung mechanics, and higher mortality than static strain ^(2)^. However, monitoring measures to assess regional ventilation homogeneity in critically ill patients at bedside are limited. Computed tomography is a standard test for examining ventilation and aeration of the lungs, but transportation of critically ill patients, radiation exposure, and limitations to static evaluation are the major obstacles for its frequent use in the clinical practice. Developing a new testing method that can be performed repeatedly at bedside is warranted.

## Electrical Impedance Tomography

**Figure 1.**
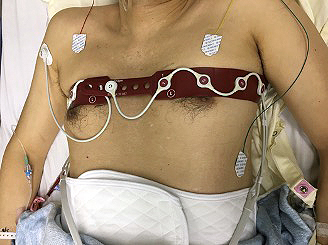
EIT belt.

**Figure 2.**
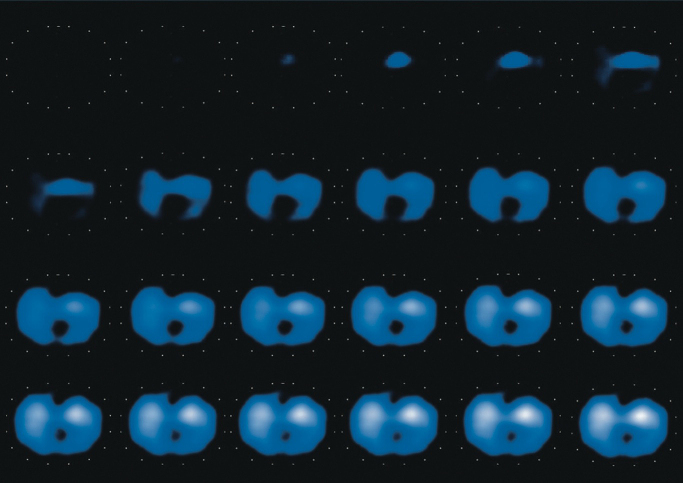
Serial static images of ventilation distribution from the start to the end of inspiration measured using electrical impedance tomography. The ventilated region is colored from blue (normally ventilated) to white (maximally ventilated), depending on the degree of the measured impedance value. Nonventilated regions are colored black. In this case, ventilation begins in ventral regions, and ventilation is distributed more to ventral than dorsal regions at the end of inspiration.

Body tissue consists of specific components such as water, lipids, and electrolytes. These components have their own responses to an externally applied electric current. Gas in the lungs acts as an electrical resistor and increases regional impedance. Ventilation causes cyclic regional changes in impedance depending on the regional difference in aeration. Electrical impedance tomography (EIT) measures and visualizes the impedance changes generated by ventilation in real time. The principle of measurement is described elsewhere ^(3)^. Briefly, the impedance value is measured by applying a small alternating current through electrodes implemented in the EIT belt, which is normally placed at the fifth to sixth intercostal level of the patient’s chest (Figure 1). Sequential cross-sectional images are reconstructed using raw impedance data, and the user watches the dynamic image of ventilated lungs. Figure 2 presents typical sequential images of EIT. With a rate of 20-30 frames per second, it is easy to recognize the spatial localization and temporal discordance or asynchrony of ventilation as a dynamic image.

EIT is radiation free, and a dynamic regional ventilation profile can be monitored at the bedside every time the ventilator settings are changed.

## Clinical Implication of EIT

EIT provides reliable information to detect alveolar overdistention and collapse. To assess the homogeneity of ventilation distribution, the patient was paralyzed, and incremental and decremental PEEP trials were performed at a fixed driving pressure (10-12 cmH_2_O). The whole procedure was monitored and recorded using EIT, and the saved impedance data were analyzed using software (EIT diag, Draeger, Luebeck, Germany).

In severe ARDS patients, EIT is used to optimize mechanical ventilation settings ^(4)^, providing useful information when titrating personalized PEEP ^(5)^. EIT might help evaluate the possibility of lung recruitment maneuvers and estimate recruitable alveolar collapse ^(6)^. Prone positioning is a rescue therapy for patients who do not respond to lung protective ventilation. We performed prone positioning for a patient with ARDS who could not maintain gas exchange with LTV and observed the process of the change in regional ventilation distribution using EIT ^(7)^. Initially, ventilation was shifted to the mid-ventral region, and the dorsal area completely collapsed. Twenty minutes after the initiation of prone positioning, ventilation distribution became more homogeneous, and oxygenation improved dramatically. The homogeneity of ventilation was stabilized after the second session, and the patient survived. Prone positioning basically provides homogeneous ventilation distribution in ARDS lungs, but temporary overdistention in the dorsal region has also been observed immediately after initiation ^(8)^. The response to prone positioning varies among cases and the close monitoring using EIT provides information to improve its efficacy.

## Roles of EIT in the Era of the Coronavirus Disease 2019 Pandemic

The outbreak of severe acute respiratory syndrome coronavirus-2 (SARS-CoV-2) is an emergent threat to the current global health. In Japan approximately 1.6% of patients with coronavirus disease 2019 (COVID-19) are severe, defined by the requirement of tracheal intubation and mechanical ventilation. It has been reported that COVID-19 pneumonia can cause ARDS. Therefore, it is necessary for COVID-19-associated ARDS to implement the same lung protection strategy described earlier. However, a recent study paid special attention to lung protection strategies for COVID-19. EIT plays a role in determining lung protective strategies for COVID-19-associated ARDS.

## Identifying Phenotypes of COVID-19 Pneumonia

There are two phenotypes that have a decisive effect on mechanical ventilation strategies in COVID-19 pneumonia ^(9)^. Conceptually, type L is characterized by low elastance, low ventilation-perfusion ratio, and low lung weight and recruitability, whereas type H is defined as high elastance, high right to left shunt, and high lung weight and recruitability. Recruitability is defined as the possibility to open up collapsed areas of the lung by positive pressure. A lung protective strategy of limiting tidal volume and plateau pressure is not required for type L but should be actively implemented in type H. However, it is not easy to distinguish between the two types because the lung weight cannot be measured clinically, and the method for evaluating recruitability is unclear. Measuring ventilation-perfusion matching at bedside has not been established. PEEP titration using EIT can not only evaluate recruitability but also determine the optimum PEEP at the time of measurement and provide regional ventilation homogeneity. As a result, it is considered possible to attenuate the regional dynamic strain and inhomogeneity of transpulmonary pressure.

To assess the phenotype, regional ventilation distribution was analyzed using EIT in mechanically ventilated patients with acute hypoxemic respiratory failure (AHRF) and ARDS due to COVID-19. Morais and colleagues ^(10)^ presented three cases of COVID-19 AHRF that had similar levels of oxygenation but variable respiratory system compliance. EIT indicated different characteristics of the regional ventilation profile and was helpful in understanding the etiology of hypoxemia at bedside. Tomasino and colleagues ^(11)^ used EIT to identify the characteristics of COVID-19 pneumonia and to decide whether to use high PEEP or prone positioning.

## Optimal PEEP

In a cohort study of mechanically ventilated non-COVID-19 patients, EIT was used to assess the optimal PEEP at which both overdistention and collapse are minimized because both phenomena were equally harmful for ARDS lungs ^(12)^.

Perier and colleagues ^(13)^ compared the response to PEEP titration between COVID-19 associated ARDS and ARDS from other causes. They defined optimal PEEP using EIT as the smallest sum of overdistention and collapse. COVID-19-associated ARDS lungs required 12 cmH_2_O of PEEP and had more collapse at low PEEP levels. Van der Zee and colleagues ^(14)^ reported that EIT monitoring during the decremental PEEP trial to seek the level of lowest relative alveolar overdistention and collapse provided personalized PEEP that did not result in high driving pressure or transpulmonary pressure. Shono and colleagues personalized PEEP levels and applied prone positioning sessions to obtain homogeneous ventilation distribution based on EIT analysis ^(15)^. PEEPset corresponded better with the higher PEEP-FIO_2_ table (used in ALVEOLI study) than the lower PEEP-FIO_2_ table (used in ARMA study), especially in patients with a higher body mass index. However, Sella and colleagues ^(16)^ compared EIT-guided PEEP and lower PEEP-FIO_2_ table-guided PEEP and found a correlation. The researchers mentioned that the opposite results might be due to differences in the interpretation of the results presented by the same device and differences in the degree of obesity.

## Ventilation-Perfusion Matching

In addition, marked hypoxemia is often observed even in cases without dorsal collapsed areas in COVID-19-infected lungs. Ventilation-perfusion mismatch is considered to be the major cause of this phenomenon. It is believed that COVID-19 hypercoagulability and vascular endothelial cell damage are responsible for this phenomenon, but the details are unclear. Because ventilation-perfusion mismatch cannot be improved by mechanical ventilation, responding to hypoxemia by increasing ventilation pressure, including PEEP, results in considerable lung injury risk due to overdistention. In summary, investigation of ventilation-perfusion matching on the bedside by EIT is useful for lung protection.

Recently, EIT has been used to assess regional lung perfusion distribution in a clinical setting. Rapid injection of a contrast agent such as 10 ml of hypertonic sodium chloride enables the generation of a lung perfusion profile during an end-expiratory hold maneuver ^(4)^. Mauri and colleagues reported that the mismatching rate was elevated to 34% (32%-45%) of lung units in COVID-19 lungs ^(17)^. Furthermore, in six of seven patients, ventilated nonperfused units (dead space) represented a much larger proportion than perfused nonventilated perfused units (shunt), although the potential for lung recruitment had large variability. The researchers concluded that an elevated dead space fraction might be a specific pathophysiological characteristic of COVID-19 ARDS.

## Preventing Patient Self-Inflicted Lung Injury

Strong inspiratory effort is often observed as a characteristic of COVID-19 pneumonia. Damaged lungs with reduced compliance generate large negative pressure in the thorax, increase transpulmonary pressure, and can cause or aggravate lung injury, patient self-inflicted lung injury (P-SILI). The pathological changes caused by P-SILI are irreversible and worsen the prognosis. Neuromuscular blockade to attenuate inspiratory drive is effective in protecting lungs against P-SILI. However, the use of neuromuscular blockade leads to alveolar collapse, especially in the dorsal regions, with inappropriate PEEP levels, resulting in inhomogeneous ventilation distribution. The efficacy of EIT to prevent P-SILI has not been tested. Further studies are warranted.

## Conclusions

This review briefly described the basic principle of EIT and summarized the latest findings on its potential contribution to lung protective strategies in COVID-19 patients. EIT is noninvasive and can be used for real-time evaluation to identify the ventilation distribution of infected lungs. The latest approach indicated the feasibility of detecting the distribution of pulmonary blood flow and evaluating ventilation-perfusion matching at bedside.

## Article Information

### Conflicts of Interest

None

### Author Contributions

Concept and design: TK. Drafting of the manuscript: TK, AS. The authors read and approved the final manuscript.
